# The Use of Fish Skin Grafts in Children as a New Treatment of Deep Dermal Burns—Case Series with Follow-Up after 2 Years and Measurement of Elasticity as an Objective Scar Evaluation

**DOI:** 10.3390/jcm13082389

**Published:** 2024-04-19

**Authors:** Raphael Staubach, Helen Glosse, Steffan Loff

**Affiliations:** Department of Pedriatic Surgery, Klinikum Stuttgart, Olgahospital, 70174 Stuttgart, Germany; h.glosse@klinikum-stuttgart.de (H.G.); s.loff@klinikum-stuttgart.de (S.L.)

**Keywords:** burns, children, wound, fish skin, Dermalab

## Abstract

**Background:** Wound healing in deep dermal burn injuries continues to be a challenge in paediatrics. In the absence quick and spontaneous wound closure, split-thickness skin grafting is often necessary. Since the development of a new type of acellular fish matrix, which is very similar to the human skin matrix, skin closure and wound conditioning can be achieved without split-thickness skin grafting. **Methods:** The following study shows a case series of 20 children in whom a fish skin graft was used. The aim was to develop an algorithm for selecting and using fish skin and its long-term results after one and two years. Acellular fish skin worked as a granulation base for wound healing and also as a substitute for split-thickness skin grafts. **Results:** There was no evidence of infection. Skin transplants and, thus, additional operations could be avoided. The follow-up examinations showed an excellent result, both objectively by means of elasticity measurements (DermalabCombo^®^) and in the subjective assessment of the skin as part of the Patient and Observer Scar Assessment Scale (POSAS). **Conclusion:** Fish skin grafts are a good alternative to split-thickness skin grafts for deep dermal wounds. These results should be further analysed with a larger number of patients in future publications.

## 1. Introduction

For deep dermal and extensive burns, early excision and split-thickness skin grafting is an established treatment option for achieving early wound closure and avoid complications such as sepsis [1]. In cases of smaller, uncomplicated residual defects, it is often possible to wait and see. A variety of wound dressings are now available to support the “wait and see” approach and ensure complication- and infection-free healing. Acellular fish skin grafts are a new option in the treatment of burns in children and have not yet been studied extensively [2,3,4]. The fish skin is made of intact skin from North Atlantic cod (*Gadus morhua*) and is processed using a proprietary method that preserves its structure and lipid composition (omega-3 fatty acids) [2]. Previous investigations state that omega-3 polyunsaturated fatty acids reduce inflammatory responses and advance proinflammatory cytokines that promote wound healing. The fish skin grafts can facilitate the transition out of the inflammatory phase of the wound healing process and for this reason accelerate the wound healing [2,5]. Thermal injuries are divided into four grades. Grade I refers only to an epidermally affected lesion without blistering. The wounds heal without consequences like scars. As soon as blisters develop in a thermal injury, it is at least a second-degree injury. The depth of the damage to the dermis results in more or less pronounced texture disorders or scarring. In this context, the fish skin graft will play an important role, as well as in smaller third-degree defects. Third-degree thermal injuries affect the entire dermis and extend into the subcutis. This injury does not heal spontaneously, and scars always occur. Surgical intervention is required for adequate treatment. In fourth-degree thermal injury, the tissue including neighbouring structures such as muscles, tendons, and bones is damaged and also requires surgical treatment or reconstruction [6,7].

The aim of this study was to demonstrate the use and clinical procedure of this new wound dressing in children. The results demonstrated that acellular fish skin grafts can be used to support the different wound healing phases and are a good alternative to split-thickness skin grafts. Furthermore, a stable skin situation with good elasticity of the scar was demonstrated using a measuring device (DermalabCombo^®^) over a period of 24 months. After treatment of the wounds, we carried out annual clinical checks to closely monitor and examine the scar quality of the affected areas. The quality of the children’s scars and their satisfaction (including that of their parents) were assessed and presented both subjectively and objectively (DermalabCombo^®^, Cortex Technology, Aalborg, Denmark); POSAS. In addition, postoperative outcomes such as the duration of wound healing and hospitalisation were evaluated.

A further aim of this work was to create a sensible algorithm for the use of acellular fish skin grafts in children and to establish a standardised procedure based on this. The focus of this innovative wound care is always on safe and optimal support of the wound bed and, above all, patient safety. All patients were treated with a new type of acellular fish skin graft from North Atlantic cod. The xenogenic wound material was used as a split-thickness skin substitute or as a dermis substitute to support wound healing.

An overview of patients with causes and extent of thermal injury is shown in Table 1. Exclusion criteria were wounds that were too superficial, a fish allergy, or burn areas that were too large (>15%KOF, III°), which would have healed faster with conventional methods such as dermis replacement (e.g., Integra). The aim was not to endanger the child at any time through the wound healing.

## 2. Materials and Methods

This study included children with deep dermal and partial subdermal with small clear third-degree thermal injuries that were necrectomised and treated early to avoid complications. A third-degree burn wound was present if fat was identified in the affected wounds of our patients. Children after thermal injury, whose wounds had been healing for two to three weeks and who were in principle indicated for split-thickness skin grafting, were also included. The depth of the wounds was confirmed and documented using a laser Doppler imaging (LDI) depth gauge (Case 2, Figure 5) [8].

The LDI penetrates the skin with the laser light and is scattered by the tissue and by the moving blood cells in the capillaries, arterioles, and venules. The moving blood cells in the tissue cause Doppler frequency shifts, which are processed to create a colour-coded map of the flow through the skin. At the same time, a camera takes a colour photo of the wound, which corresponds exactly in size and appearance to the image of the blood flow. The measurement is non-contact and can quantify differences in blood flow over a specific area of tissue and thus provide information on depth [9].

The different methods used in these examinations make direct comparisons between different studies difficult and often make it difficult to interpret the results accurately. Therefore, efforts to introduce standardised procedures and improve examinations are crucial to gain clearer and more reliable insights into the mechanical properties of the skin [10,11].

Therefore, we used the DermalabCombo^®^ skin elasticity measuring device in our study, also due to its ease of use. The retraction time was measured in milliseconds. The longer the retraction time, the higher the elasticity. To support this, the skin thickness was determined in micrometres using ultrasound.

### 2.1. Algorithm

**Figure 1:**
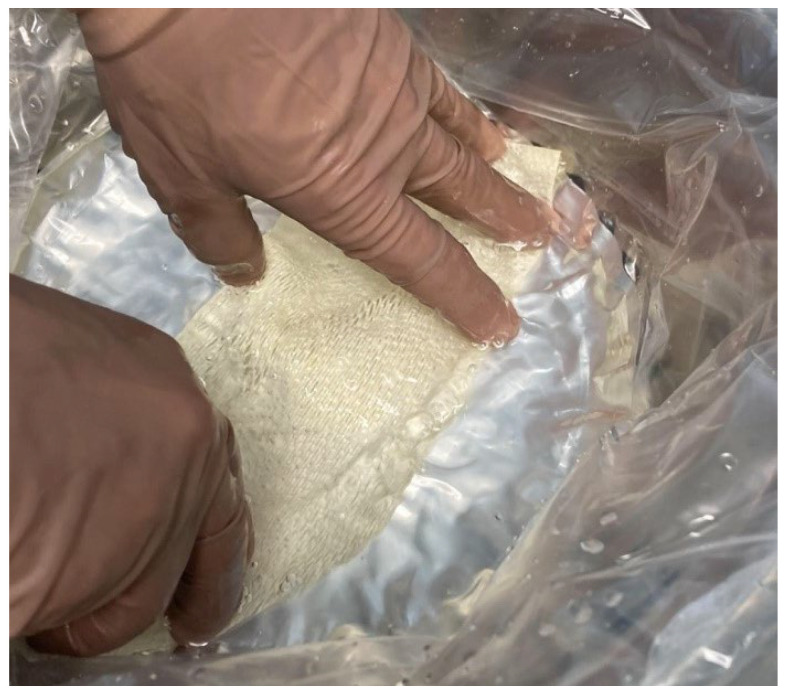
Fish skin graft soaked in NaCl 0.9%.

**Figure 2:**
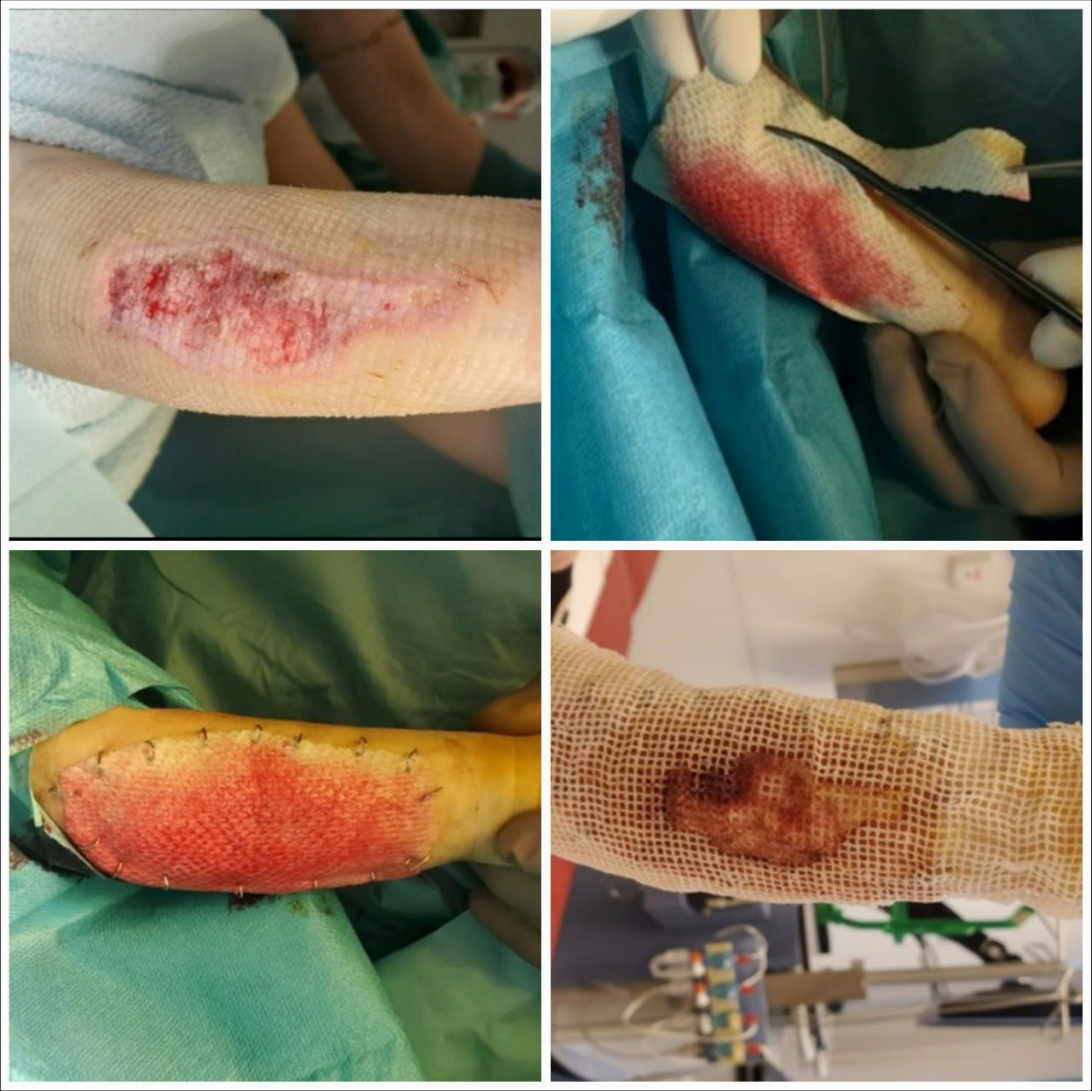
Short debridement and application of a fish skin graft.

In the operating room, deep, non-healing wounds were hydrosurgically debrided using Versajet. Versajet uses a saline jet to grasp, cut, and remove the necrotic tissue while preserving viable tissue for effective wound bed preparation. Meanwhile, an acellular fish skin graft soaked in NaCl 0.9% (Figure 1) and meshed 1:1 or 1:2 was then applied and fixed with staples (Figure 2) or sutures. Subsequent dressing changes could then be performed painlessly every 2–3 days in an outpatient setting. Larger third-degree areas covered with fish skin grafts were kept moist using Prontosan (polyhexanide) and a fat-containing wound covering (e.g., Bacticras). This allowed the graft to integrate into the wound to support and accelerate wound healing. The aim was to reduce the size of the large wounds and, if necessary, to condition them for further measures.

**Figure 3:**
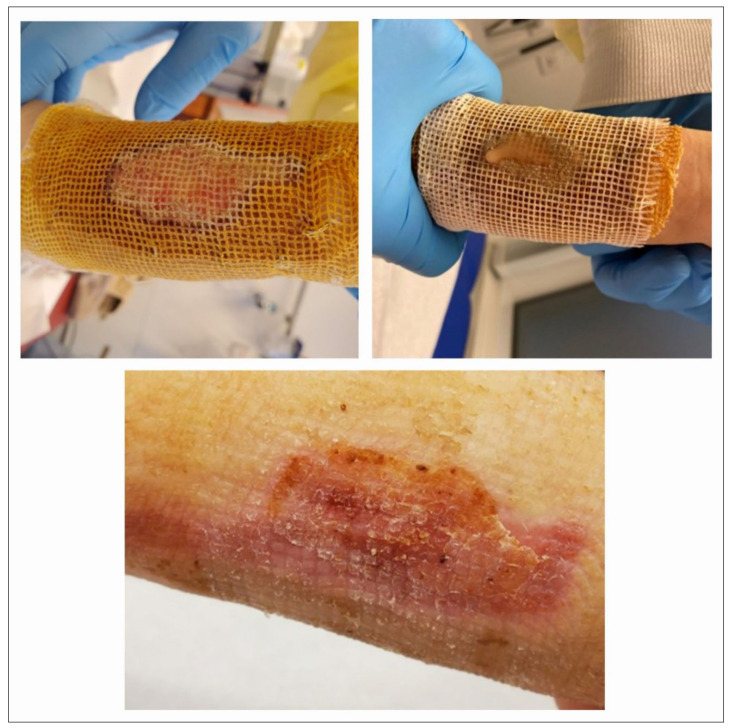
“Active slime” and the result after 22 days.

Meanwhile, in smaller areas, the fish skin graft was kept dry and used more as a carrier for cell integration and wound sealing. Here, the patient’s own wound secretion was usually sufficient to keep the xenogenic material moist. Dressings were also changed every 2–3 days (Figure 3), using Octenisept and a polyhexanide solution, for example, and usually in an outpatient setting.

### 2.2. Clinical Follow-Up

The quality of the children’s scars and their satisfaction (including that of their parents) were examined in more detail using the DermalabCombo^®^ skin measurement device (elasticity measurement and visualisation of thickness and structure using sonography) and also evaluated using a patient-oriented score (“Patient and Observer Scar Assessment Scale”, POSAS). The DermalabCombo^®^ examination was performed on the affected area and on healthy skin on the opposite side of the body. The aim here was to visualise the scar quality after fish skin application.

The POSAS assessments were carried out at each clinical follow-up. In the POSAS assessment, the affected region was compared to the healthy skin. The scar was assessed by the examiner, the examining doctor, and the patient or their parents. The assessment was carried out independently. Points were awarded from 1 to 10, with 10 being the worst score. The examiner assessed the vascularity, pigmentation, thickness, relief, and smoothness, as well as the extent, and awarded corresponding points. The patient assessed factors such as pain and itching of the scar, colour, stiffness, thickness, and relief.

Other factors such as postoperative complications, the duration of wound healing, and hospitalisation were also assessed.

### 2.3. DermalabCombo^®^ Skin Analysis

DermalabCombo^®^ is a skin testing device developed by Cortex Technologies in Denmark to measure the skin in a standardised way. Although it is primarily aimed at cosmetic applications, it is increasingly being used in the medical field [12]. The different probes cover several functions. However, in this study, the two most established skin analysis technologies were used to assess the skin status of patients—the ultrasound and the skin elasticity probe [12,13,14].

### 2.4. Ultrasound Skin Analysis

Ultrasound skin imaging is based on the detection of sound responses emanating from both the skin and subcutaneous tissue. The emitted sound pulse hits various skin structures. Part of it is reflected, while another part penetrates further into deeper tissue.

The intensity of the received signal is represented by a colour, with dark colours representing areas with minimal reflection, indicating a homogeneous composition. Conversely, lighter colours indicate regions with strong reflections, indicating an inhomogeneous composition and significant differences in structural density [12].

**Scheme 1:**
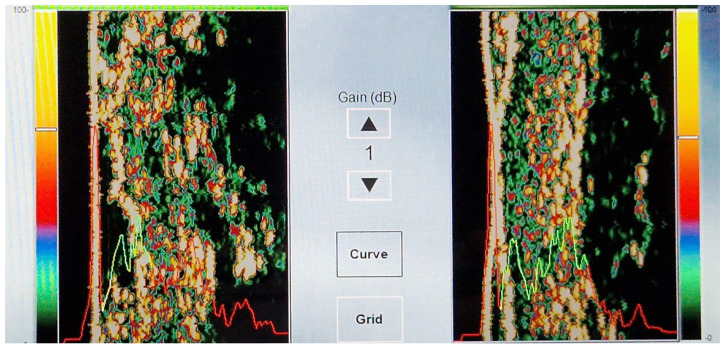
Ultrasound.

The ultrasound image starts with the epidermis on the left side, followed by the dermis and the subcutis. The dermis shows different intensities, which are reflected in its different colours, while the subcutis shows areas of low intensity due to its homogeneous composition (Scheme 1) [12].

### 2.5. Technical Data

The probe for measuring skin thickness was equipped with a high-frequency ultrasound probe operating at 20 MHz and enabled a penetration depth of 3.4 mm. This probe had an integrated rotating ultrasonic transducer that effectively scanned a diameter of 17 mm [11,15].

### 2.6. Ultrasound Measurements

**Scheme 2:**
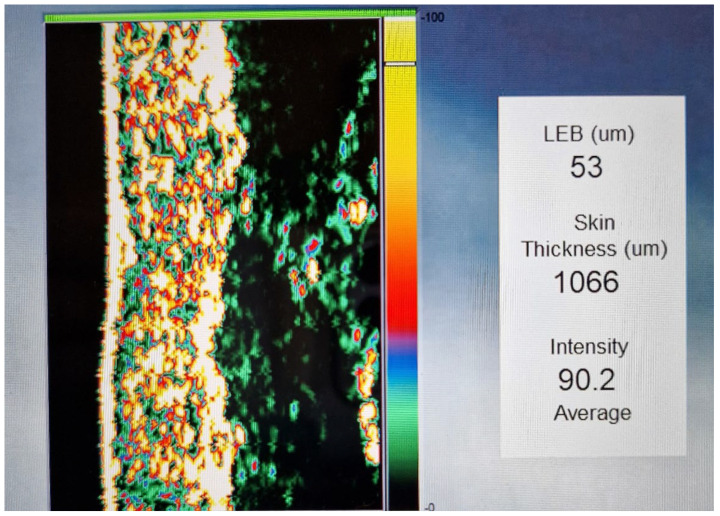
Skin thickness.

In addition, the ultrasound image provides so-called “curve” measurements, which are visible as a red/yellow/green curve. The red sections indicate the intensity through the water chamber of the probe, with the left peak coming from the reflection of the skin/epidermis. The red section on the right is the intensity through the subcutaneous layer. Yellow represents the low-echogenic band (LEB) of the dermis, which may occur due to light ageing. Green indicates the reflective deeper dermis. In combination, the red and green curves together represent the average skin thickness (Scheme 2).

Diseased skin may show different ultrasound patterns, with uniform areas appearing dark and non-uniform structures showing different reflections due to altered acoustic properties.

### 2.7. Analysing Skin Elasticity

The elasticity measurements were based on a negative pressure applied to the skin surface. A negative pressure of 150/400/650 mbar, which reflects the soft/normal/firm settings, was applied to the skin area being analysed. The lifting and retraction phases of the skin were recorded. These two parameters contribute to the functionality of the skin [12].

Young and smooth, soft skin that is well moisturised, for example, can normally be lifted relatively easily by suction and retracts quickly. Old and sagging skin is also easy to lift but does not retract quickly. Therefore, skin elasticity is usually considered a complex phenomenon that can be measured by taking into account the lifting and retraction phases [12]. SkinLab Combo provides three descriptive parameters for skin elasticity: Young’s modulus of elasticity (E), the skin retraction time (R), and viscoelasticity (VE).

### 2.8. Young’s Modulus of Elasticity (E)

Young’s modulus of elasticity (E) is an important mechanical attribute that characterises the stiffness of a material under tensile or compressive forces. It quantifies the relationship between stress (σ), which indicates the force per unit area, and strain (ε), which represents the proportional deformation in the range of linear elasticity [13].

$$E=\frac{\sigma }{\varepsilon }$$

### 2.9. Skin Retraction Time (R)

The retraction time (R) is defined as the time in milliseconds it takes for the skin to retract from its peak height to 33% of that peak height. This metric is displayed in the red segment of the elasticity diagram [12].

### 2.10. Viscoelasticity (VE)

Dividing Young’s modulus by the retraction time yields a parameter called viscoelasticity (*VE*), which considers both the lifting and retraction phases. *R* is normalised by a retraction time of 260 ms (this corresponds to the normal *VE* in 28–60-year-olds) [12].

$$VE=\frac{E}{R}$$

### 2.11. Elasticity Probe

Elasticity measurement with DermalabCombo^®^ was carried out by vertical suction directly on the skin surface. To ensure accurate measurements, the probe is placed on the skin with an adhesive. The probe contains a central 10 mm circular opening. Inside this probe are two infrared sensors, placed 1 and 2.5 mm above the skin surface, which register the elevation and retraction of the skin, achieved by the controlled application of a DermalabCombo^®^-generated vacuum. The latter is connected to the probe via a connecting cable. The modulus of elasticity is calculated by determining the differential force required to lift the scar or skin surface by 1.5 mm. This process takes place between the two infrared detection planes located in the probe chamber [12].

### 2.12. Measurement of Elasticity

Three repetitive cycles were performed to measure skin elasticity. Three cycles were used to obtain a validity check for the correct application of the probe to the skin. However, repeated measurements inevitably led to a gradual increase in the rise and retraction time with each cycle, resulting in a slight decrease in E and VE after each examination [12].

## 3. Results

The average age of the 20 patients was 8 years (Table 1, mean and standard deviation 7.6 and 4.6). In each case, careful wound debridement was performed and an acellular fish skin graft was applied. Subsequently, a rapid discharge from the hospital was possible for 60% of cases (n = 12) on the same day. Rapid and infection-free wound healing was observed in all patients, with a wound surface coverage of 95% after an average of 26 days. The operation time was, on average, 34 min, without complications or allergic reactions. Further dressing changes until healing were virtually painless and therefore not traumatising for the patients. The patients were treated according to the algorithm described above.

After the wounds had healed, our aim was to observe the condition of the scars after treatment with fish skin in the further course, particularly with regard to the tendency of scars to shrink and contract. All patients showed stable and pliable skin at the 1- and 2-year follow-ups (see case studies 1 and 2), confirmed by the elasticity measurement and visualisation with the ultrasound probe. Antibiotic coverage was only carried out on initially clearly infected wounds (n = 1, 5% of all patients). Compression treatment and silicone and ointment therapy were also carried out consistently in all patients, in accordance with the guidelines [16].

### 3.1. DermalabCombo^®^

**Scheme 3:**
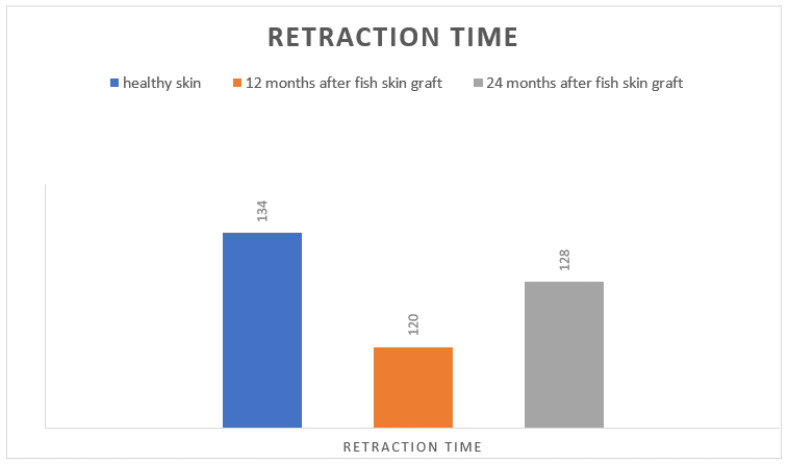
Measurement elasticity of healthy skin and scars after the use of fish skin grafts.

The modulus of elasticity of DermalabCombo^®^ showed almost identical average retraction time values compared to healthy skin one and two years after the accident. The retraction time of the healthy skin was 134 ms (standard deviation 4.9), while that of the affected scar was 120 ms at 12 months (standard deviation 5.0) and 128 ms after 24 months (standard deviation 3.1; Scheme 3).

The ultrasound module (DermalabCombo^®^) was used to determine the skin thickness and structure. The average skin thickness was found to be 900–1200 µm. The overall structure of the dermis could be uniformly visualised.

### 3.2. POSAS

**Scheme 4:**
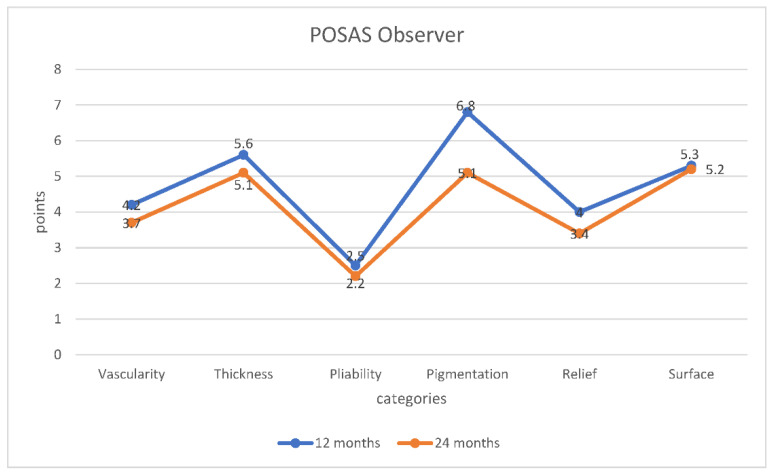
POSAS observer.

**Scheme 5:**
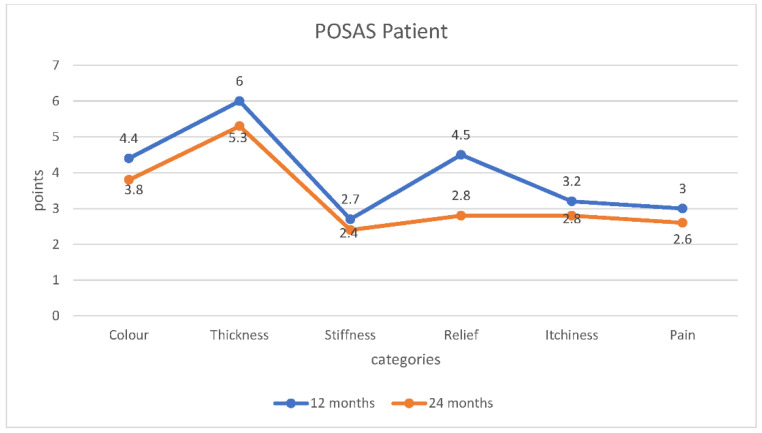
POSAS patient.

On average, the POSAS showed a decreasing score by both the observer and the patient and thus a better condition of the scar (see Scheme 2 and Scheme 3). Assessment of the thickness of the area treated with an acellular fish skin graft compared to healthy skin by the patient and their parents showed a decreasing value to 5.3 points from an initial score of 6 points, thus resulting in a better appearance. Similar findings were also observed by the examiner. The initial assessment was 5.6 points, which then fell to 5.1 points over the course of the study. The assessment of vascularity was also striking. After 12 months, the average score of the patients was 4.4 points, and after 24 months, it was 3.8 points. The investigators’ evaluation showed similar results. Initially, the average score was 4.2 points, decreasing to 3.7 points over time. The aspect of stiffness or suppleness of the affected area was rated as very good by both the examiner and the patient right from the start, with an average of 2.7 points for the patient and 2.5 points for the examiners. After 24 months, the suppleness score showed very good results, with an average score of 2.4 (patient) or 2.2 (examiner) (Scheme 4 and Scheme 5).

## 4. Case 1

A 6-month-old infant in a palliative situation. The parents did not want surgical intervention. An initial brief debridement and application of a fish skin transplant were carried out (Figure 2). The results after 3 weeks with classic “active slime” and the clinical healing results are shown in Figure 3. The “active slime” represents a physiological connection between the fish skin and the wound secretion and should not be interpreted as an infection.

## 5. Case 2

**Figure 4:**
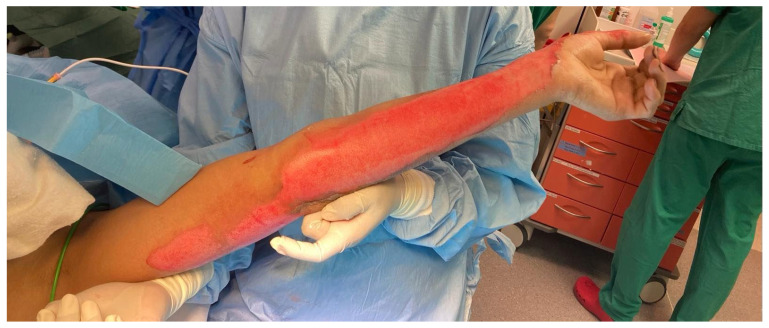
Left arm deeper dermal burn, 4 h after injury.

**Figure 5:**
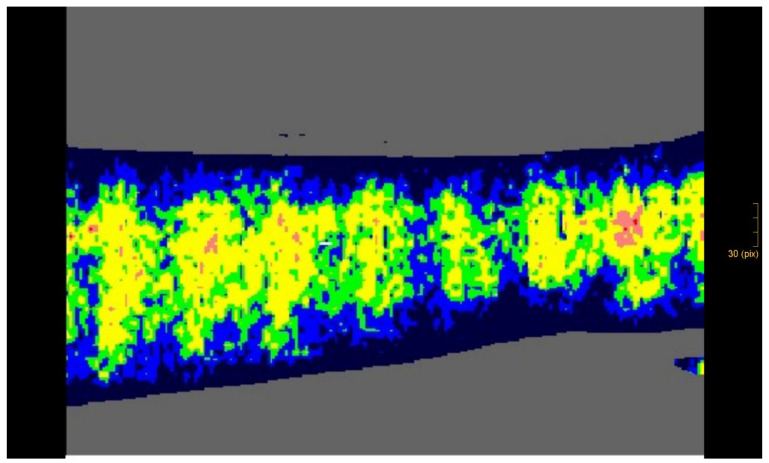
“Laser Doppler Imaging” (LDI).

**Figure 6:**
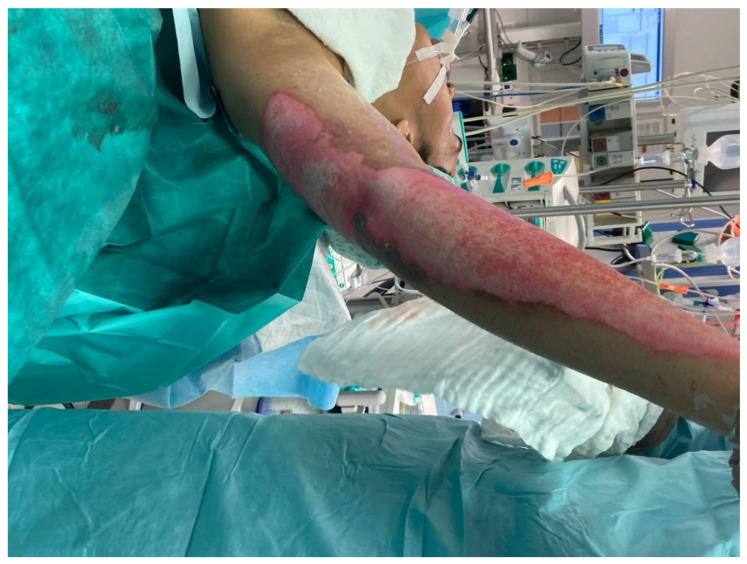
Left arm after application of Nexobrid (enzymatic debridement).

**Figure 7:**
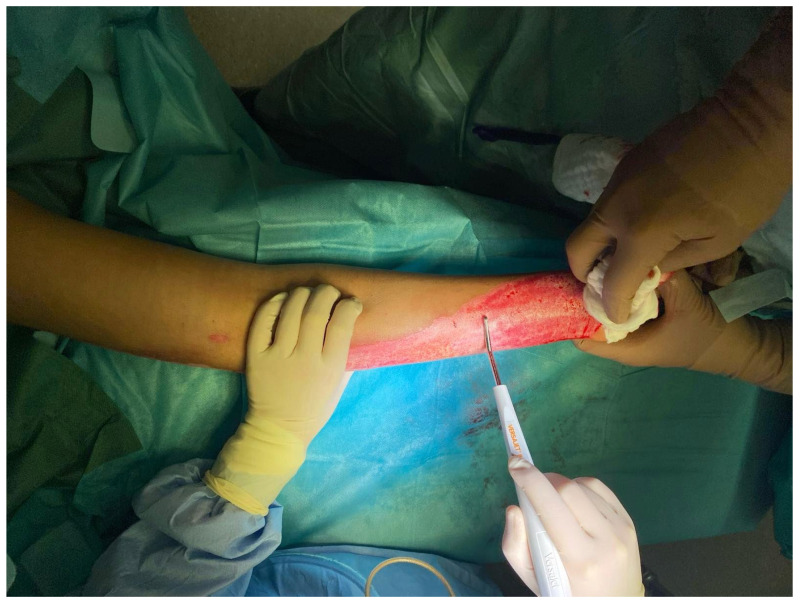
Short debridement with Versajet before fish skin application after post-soaking.

**Figure 8:**
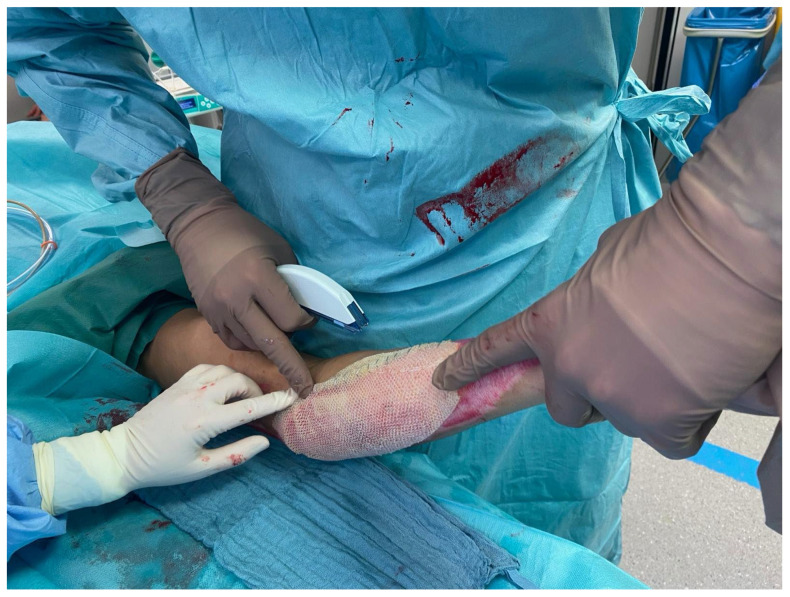
Application of a fish skin graft.

**Figure 9:**
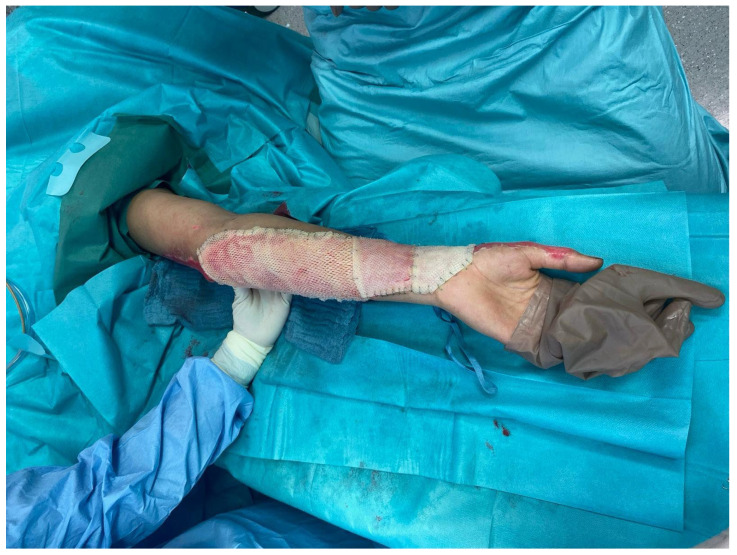
A fish skin graft.

**Figure 10:**
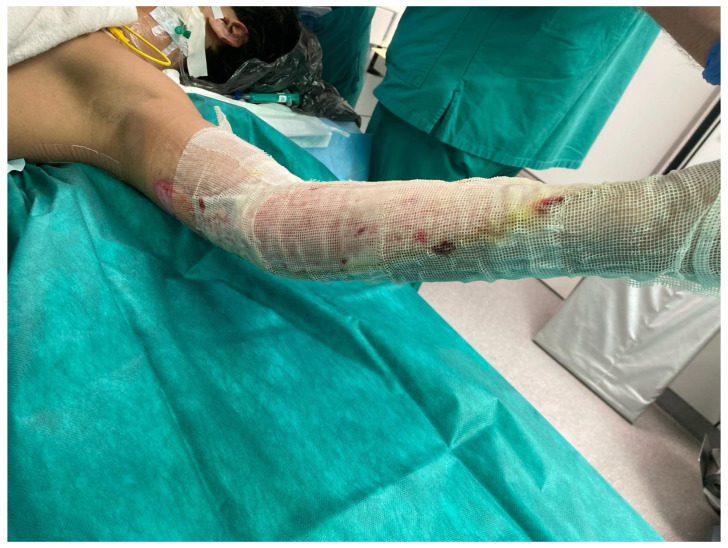
Dressing change 5 days after application.

**Figure 11:**
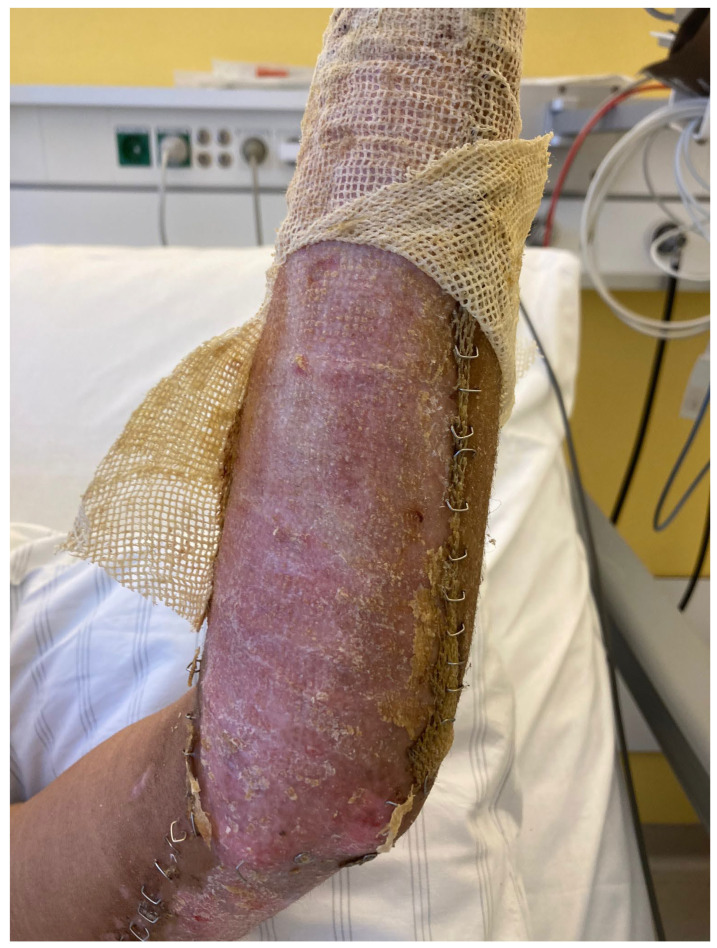
Results after 3 weeks.

A 15-year-old patient after deflagration of the extremities (Figure 4, 4 h after injury). Measurement was conducted using LDI (Figure 5), with application of Nexobrid after 48 h. After removal of Nexobrid, the area was briefly freshened with Versajet (Figure 6 and Figure 7), then the fish skin graft was applied after 72 h (Figure 8, Figure 9 and Figure 10). The healed wound situation in the partly third-degree areas after 3 weeks is shown in Figure 11.

## 6. Case 3

**Figure 12:**
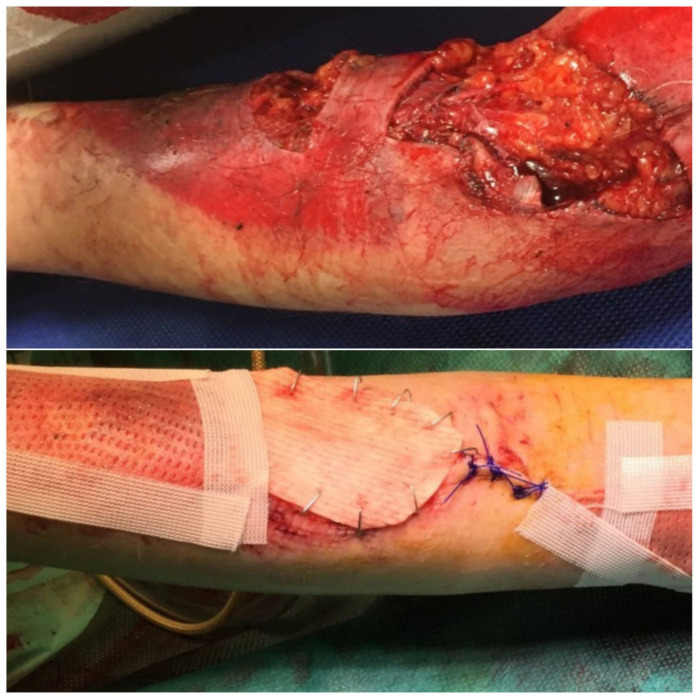
Injury left arm and fish skin graft after 10 days.

**Figure 13:**
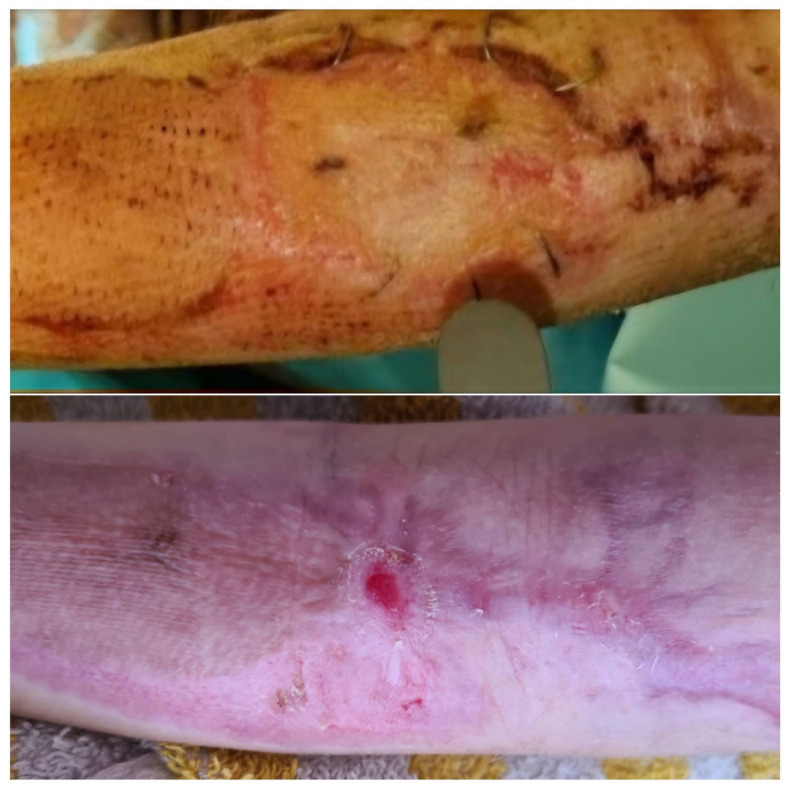
Left arm 2 and 4 weeks after injury.

**Figure 14:**
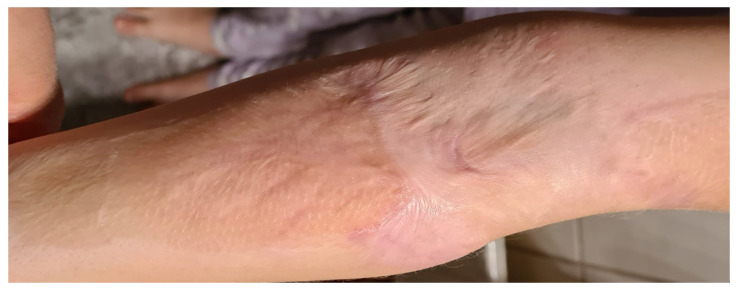
Left arm 2 years after injury without contractions.

An eight-year-old girl after trauma with extensive injuries to both legs and left arm. A deep defect after leathering was treated with the acellular fish skin graft after 10 days, in a clean wound situation (Figure 12). New dressing after 2 weeks and closed wound after 4 weeks (Figure 13). No further transplantation was necessary. The healed area, which was treated with the acellular fish skin graft, shows only minor hypertrophic scar areas and is also very similar to healthy skin in terms of elasticity and thickness measurement. In Figure 14 (2 years after injury), wound healing can be seen in the manner of secondary wound healing, supported by the fish skin graft as a carrier and possibly as an induction for faster and safer wound healing. Due to major injuries and their treatment on both legs, we decided to graft the left arm with fish skin graft.

## 7. Conclusions

The application of fish skin grafts represents an innovative and sustainable treatment option in paediatric wound care, particularly for deep dermal or small third-degree thermal injuries. This case series is one of the largest in which acellular fish skin grafts were used to treat paediatric patients following deep dermal wounds. However, it must be added that the size of the study group and the lack of a control group, which is generally a problem when analysing scars, should be noted.

The focus is on providing omega-3 fatty acids and promoting interactions of interleukins for infection-free and supportive wound healing. Studies have shown that omega-3 polyunsaturated fatty acids reduce inflammatory reactions and promote proinflammatory cytokines, which support and accelerate wound healing [17,18]. Acellular fish skin grafts containing these polyunsaturated omega-3 fatty acids can therefore facilitate the transition from the inflammatory phase to the regeneration phase of the wound healing process [19,20].

Fish skin for chronic wounds, which is already established in vascular surgery, can also find its place in burn treatment [17]. Fish skin is also already highly valued in adult burn care [21]. In our experience, fish skin is a sensible and uncomplicated alternative for residual defects after, for example, a classic bib burn. The healing of these areas, which were, in principle, intended for split-thickness skin grafting, was more than satisfactory in terms of scar quality and cosmetic outcome (see case series).

In addition, the use of fish skin leads to a significantly shorter operation time compared to a split-thickness skin transplant and, therefore, also to shorter hospital stays. The number of split-thickness skin transplants can potentially be minimised through the individual use of fish skin. However, fish skin cannot be used as a direct substitute for split-thickness skin transplantation—especially for large areas—but it can be a useful wound dressing intermediate between temporary wound dressings and a definitive split-thickness skin transplant. With an acellular fish skin graft, it is possible to support conservative wound healing or to achieve sufficient conditioning prior to possible split-thickness skin grafting, so that an aseptic inflammation-free wound with as much regenerated or preserved dermis as possible is available.

In children in particular, it is essential to find the right balance between radical and conservative treatment with the number of dressing changes and to keep these as low as possible in order to avoid traumatising the children or performing general anaesthesia too frequently. The aim is also to close wounds as quickly as possible but also without overly generous split-skin indications. According to our algorithm, we recommend changing the dressing every 2–3 days under sterile conditions. For smaller wounds, the fish skin graft can be disinfected, e.g., with Prontosan or Iodoform in combination with a fat gauze, and kept dry. For larger wounds, the fat gauze should be left in place and moistened every 2–3 days (e.g., Prontosan). After 3 weeks, the fat gauze can be removed, and further care can be provided according to the wound healing phase.

In conclusion, it can be said that the method could possibly minimise the number of split-skin indications in the future without traumatising the child due to excessively long wound healing. Using the fish skin graft reduces the operation time, eliminates the need for donor sites for split skin indications, and drastically reduces the length of hospitalisation.

Fish skin obtained from cod contains collagen, fibrin, proteoglycans, and glycosaminoglycans and, according to current studies, acts as a skin substitute [17,18]. Whether marine fish skin can compete with already proven materials as a dermis substitute, particularly with regard to the resulting skin texture, must be investigated in detail in future studies. One possible study variant is the bioptic examination of a wound treated with fish skin in comparison to clinical controls.

## Figures and Tables

**Table 1:** Clinical data.

Sex	Age	TBSA in %	3rd Degree	Reason	Epithelisation in d	Retraction Time Healthy	Retraction Time after 1 Year	Retraction Time after 2 Years
m	0.5	1	0.5	Boiling water	33	150	125	134
m	15	10	3	Explosion	20	130	117	124
m	8	0.5	0.25	Electricity	23	135	115	124
w	15	1.5	1	Oil	21	130	128	130
m	1	5	1.5	Boiling water	20	138	125	133
m	1	1	0.25	Contact burn	30	137	114	127
w	9	2	1	Oil and water	27	130	115	124
m	3	1	0.6	Contact burn	24	132	122	125
w	8	5	2	Boiling water	28	134	119	130
m	2	2	0.3	Oven	28	134	123	128
w	14	2	1	Ice spray	24	138	115	127
m	12	1.5	0.6	Explosion	29	132	118	124
m	11	1	0.7	Friction	20	133	122	129
w	10	2	1	Contact burn	26	139	125	130
w	9	4	2.5	Boiling water	28	130	118	127
m	8	2.5	1.5	Boiling water	27	132	114	126
w	10	3	0.4	Burn	28	129	113	124
m	4	5	2	Boiling water	22	138	126	130
m	12	2	1	Explosion	27	130	128	128
w	4	3	0.8	Boiling water, oil	29	139	127	132

## Data Availability

Data is contained within the article.
